# Results of the Chronic Heart Failure Intervention to Improve MEdication Adherence (CHIME) Study: A Randomized Intervention in High-Risk Patients

**DOI:** 10.1016/j.ahj.2015.01.006

**Published:** 2015-01-14

**Authors:** Bradi B. Granger, Inger Ekman, Adrian F. Hernandez, Tenita Sawyer, Margaret Bowers, Tracy DeWald, Yanfang Zhao, Janet Levy, Hayden B. Bosworth

**Affiliations:** 1 Duke University School of Nursing, Durham, NC; 2 Duke University Health Systems, Durham, NC; 3 Institute of Health and Care Sciences, Centre for Person-Centred Care, University of Gothenburg, Göteborg, Sweden; 4 Duke Clinical Research Institute, Duke Medicine, Durham, NC; 5 SAS Institute, Cary, NC; 6 Center for Health Services Research in Primary Care, VA Medical Center Durham, NC

**Keywords:** medication adherence, symptoms, heart failure, self-care, patient education, meaning response, beliefs

## Abstract

**Background:**

Poor adherence to evidence-based medications in heart failure (HF) is a major cause of avoidable hospitalizations, disability, and death. To test the feasibility of improving medication adherence, we performed a randomized proof-of-concept study of a self-management intervention in high-risk patients with HF.

**Methods:**

Patients with HF who screened positively for poor adherence (<6 Morisky Medication Adherence Scale 8-item) were randomized to either the intervention or attention control group. In the intervention group (n=44), a nurse conducted self-management training prior to discharge that focused on identification of medication goals, facilitation of medication-symptom associations, and use of a symptom-response plan. The attention control group (n=42) received usual care; both groups received follow-up calls at 1 week. However, the content of follow-up calls for the attention control group was unrelated to HF medications or symptoms. General linear mixed models were used to evaluate the magnitude of change in adherence and symptom-related events at 3-, 6-, and 12-month follow-up clinic visits. Efficacy was measured as improved medication adherence using nurse-assessed pill counts at each time point.

**Results:**

Pooled over all time points, patients in the intervention group were more likely to be adherent to medications compared with patients in the attention control group (odds ratio [OR] 3.92, t=3.51, p=0.0007).

**Conclusions:**

A nurse-delivered, self-care intervention improved medication adherence in patients with advanced HF. Further work is needed to examine whether this intervention can be sustained to improve clinical outcomes.

Chronic heart failure (HF) remains a leading cause of disability and death in adults.^1^ Poor adherence to approved, evidence-based medications accounts for the majority of preventable HF hospitalizations and deaths.^2–4^ Large community-based clinical trials have shown that medication adherence is associated with improved outcomes.^5,6^ Yet patients struggle with taking medications as prescribed, and adherence rates remain stagnant at around 50%.^7,8^ Many factors increase the risk for poor adherence; chief among them for patients with HF is symptom persistence resulting in loss of belief in medications.^9,10^ Recurrent and persistent symptoms in HF such as fatigue and orthopnea are associated with poor adherence and premature or unnecessary discontinuation of medications. ^11,12^

Interventions such as coaching,^13,14^ education,^15^ and reminder strategies^16^ have been developed to improve adherence to medications.^17,18^ Most focus on educational content delivery and yield only short-term success,^19^ though some with more intense personal contact have shown improvement in adherence and clinical outcomes (readmissions, mortality, emergency department visits) at 12 months.^20,21^ Despite isolated success, the prevalence of poor adherence and premature medication discontinuation persists.^22^

A new approach to closing the symptom-adherence gap is tested in this study and is based on Meaning Response Theory (MRT).^23^ Meaning-response constructs add to the existing theories^22–24^ that explain our current understanding of medication-taking behavior by introducing intentional connections between medications and the symptom-response. These associations may be used to reframe medication-taking from “burden” and “work,” to shared responsibility and partnership with care providers.^25^ Helping patients reframe medication-taking in this way may build positive associations with medications and may help patients perceive adherence as a daily health achievement rather than an effort to reach unattainable symptom resolution.^26,27^ Developing positive associations with a treatment regimen has been shown to be associated with better short-term adherence.^28,29^ Interventions that use meaning-associations to coach patients to respond quickly and appropriately to symptom changes may improve medication adherence, particularly in the context of chronic symptom exacerbation. However, meaning-association interventions have not been evaluated in chronic HF.

We conducted preliminary work to explore the theoretical linkages between symptom experiences^29^ and meaningful associations with medications.^30^ The purpose of this study was to evaluate the clinical efficacy of a new intervention for improving medication adherence. The CHIME 3-M (Chronic Heart Failure Intervention to Improve Medication Adherence Medications, Meaning, and Me) intervention was designed to help patients with chronic HF develop meaningful associations with medicines with the goal of improving medication adherence.

## METHODS

A prospective, randomized, controlled design was used to test the efficacy of the proposed intervention among patients admitted to a large southeastern academic medical center with a primary diagnosis of HF. Patients were identified and pre-screened for poor adherence using a validated screening measure, the Morisky Medication Adherence Scale (MMAS 8-item).^31^ Of those patients who initially consented to participate (n=265), 165 patients scored “usually adherent” (MMAS 8-item score ≥6) at baseline and were not eligible. One patient withdrew consent prior to randomization. The remaining patients (n=86) were randomly assigned to either the intervention (n=44) or attention control (n=42) group. The attention control group received usual care and had contact with the nurse at the same frequency as the intervention group, but with scripted health promotion messages that were not related to medications or HF symptoms. The intervention was conducted in the hospital setting prior to discharge, patients received a follow-up phone call at 1 week, and booster intervention activities took place at the clinic during standard follow-up visits at 3, 6, and 12 months. The efficacy of the intervention, improved medication adherence, was measured as nurse-assessed pill counts (primary endpoint). In addition, secondary measures included patient-reported medication adherence (measured using the MMAS 8-item), patient-reported belief in medications (measured using Horne’s Belief in Medications Questionnaire [BMQ]^32^), and patient-reported use of the symptom-response plan.^25,30,33^ These measures were evaluated in both groups, with the exception of the symptom-response plan log, at 3, 6, and 12 months.

### Sample and sample size calculation

In addition to being admitted with a primary diagnosis of HF and screening positive for poor adherence (MMAS 8-item score <6), criteria for participation included having a telephone for follow-up calls and having New York Heart Association (NYHA) class III IV HF for at least 6 months prior to the acute admission. This criterion was included because medication adherence in the first 6 months is usually high and the intervention is unlikely to have additional benefits in the early months following initial diagnosis. Exclusion criteria included the presence of congenital HF etiology, and an inability to provide self-care, including nursing home residents and patients requiring full-time at-home assistance, since these patients would not be able to carry out the intervention.

The sample (n=86) was sufficient for detecting a 25% difference in adherence rates between the intervention and attention control groups at a power level of 85% and a (2-tailed) type I error rate of 0.10. Since this is the first longitudinal evaluation of the intervention, we were willing to accept a larger type I error rate in exchange for more power to detect effects, if present. We evaluated differences in actual participants as compared with those who refused to participate in the study by obtaining information from charge nurse reports and medical record review. We found no significant differences that would indicate non-participation bias.

### Intervention

The initial development and feasibility testing of the intervention has been previously reported.^30^ We used patient perspectives obtained using mixed methods (e.g., qualitative and quantitative surveys) and responses to develop 3 intervention components. These components addressed both the intrinsic, cognitive understanding of medications, and the extrinsic, behavioral response associated with that understanding. Our 3-component intervention framework medication bundles, symptom triggers, and the symptom-response plan was designed to support medication adherence in situations leading to loss of belief in the necessity for medications and increasing risk for poor adherence. The components of the CHIME 3-M intervention focus patient-provider work on “Medications, Meaning, and Me” as follows: 1) Medications: facilitating skill-based learning of the medication regimen using a pill box tool; 2) Meaning: establishing medication concerns and perceived necessity of medications and establishing meaningful associations between medication adherence and symptom onset, for both patients and family caregivers, by helping patients identify symptom triggers; and 3) Me: developing a person-centered response by using symptom triggers to prompt a rapid, planned response to early symptom exacerbation.^25,33^

This study tested the hypotheses that poorly adherent patients with symptomatic HF who received the intervention would be more adherent to HF-related medications during the 12-month intervention as compared with an attention control group.

### Measures

Hypothesized outcomes for each component of the intervention were measured with both an observed and subjective (patient-reported outcome questionnaire) measure (Table 1). The primary outcome, medication adherence, was measured using nurse-assessed pill counts. For the nurse-observed assessment, we assessed the pill count for all medications because we were interested in evaluating medication management as a general skill that is required not only for one indication or illness, but in HF, for many medications due to concomitant illnesses. The nurse observed all pill bottles, pens, and vials and recorded whether or not the patient had been adherent at least 80% of the time for each prescribed medication. If the patient had taken ≥80% of each of the medications, they were ascribed a categorical classification of “adherent.” If the patient was non-adherent (<80% of medication actually taken) for at least 1 medication, then the patient was considered non-adherent.

HF-specific medications were assessed as a secondary measure using the MMAS 8-item questionnaire. For the MMAS 8-item, we specified HF medications by using the stem “heart failure” rather than “blood pressure” as in the original MMAS 4-item (Cronbach’s alpha for this cohort: 0.62–0.72). The patient also completed study questionnaires to assess patient-reported beliefs in medications (BMQ), and symptom frequency and intensity at each encounter (baseline, 3, 6, and 12 months). At baseline, the nurse presented and explained to the patient each component of the intervention and conducted the baseline pill count.

### Procedures

After identification of eligible participants, the nurse obtained informed consent to assess medication adherence and administered the MMAS 8-item to identify patients likely to be poor adherers.^8,9^ For patients likely to be poorly adherent (MMAS 8-item score <6), the nurse obtained informed consent for participation in the remainder of the study. If the patient did not have difficulty with medication adherence (MMAS 8-item score ≥6), the individual was not enrolled. After screening, patients were randomized to intervention or attention control using a block randomization strategy.

#### Intervention group

A detailed training manual for the intervention was developed for the nurse interventionist based on the components of the intervention developed in the pilot phase.^30^ Following randomization and prior to discharge, patients in the intervention group participated in an in-depth, semi-structured interview to ascertain beliefs, concerns, and perceived necessity of the prescribed medication regimen. Using patient-reported concerns and perceived necessity of medications, the nurse developed a symptom-response plan with the patient and caregiver (Table 2). This plan focused on decision-support for activation of family and community resources for symptom verification and transportation. Patient-specific symptom triggers were recorded and the patient was coached to use these triggers to prompt an early response to symptom exacerbation. Next, the nurse reviewed with the patient instructions on how to use each component of the intervention. Written instructions were provided to participants’ caregivers to reinforce the verbal instructions, and a telephone contact number for the study team was given to allow patients open access to the nurse. At each usual clinic visit following enrollment (3, 6, and 12 months), the nurse reviewed with the patient the intervention components. Any difficulties using the scales, pill boxes, calendars, and symptom-response plans were monitored and reviewed weekly by the investigative team. The patient was coached to activate the symptom-response plan if they recognized a need to intensify symptom management or add supplemental medications such as diuretics during a symptom exacerbation.

#### Attention control group

Patients randomized to the attention control group received usual care and had the same frequency and duration of contact time with the nurse as those in the intervention group (follow-up phone call at 1 week and clinic visits at 3,6, and 12 months). The content for each “contact session” focused on scripted discussion of content unrelated to HF, medication adherence, or HF symptoms. Standard care included a physical examination by the care provider, review and adjustment of medications if necessary, and review of recommendations for sodium restriction and daily weight monitoring. Patients also received the standard HF patient education notebook that was given to all patients.

### Analysis

Group differences (intervention vs. attention control) on potential confounders were tested and baseline differences in sex and living alone were identified, with females being more prevalent in the intervention group. Responders and drop-outs were also compared on independent and dependent variables. We used generalized linear models to test the primary and secondary hypotheses. Logistic generalizations of the traditional, multivariate generalized linear models were used to evaluate the dependent variable, a binary measure of the probability of medication adherence. Results were analyzed by intention-to-treat and data for adherence were censored at the time of death. Lost to follow-up was considered non-adherent.

## RESULTS

**Figure 1:**
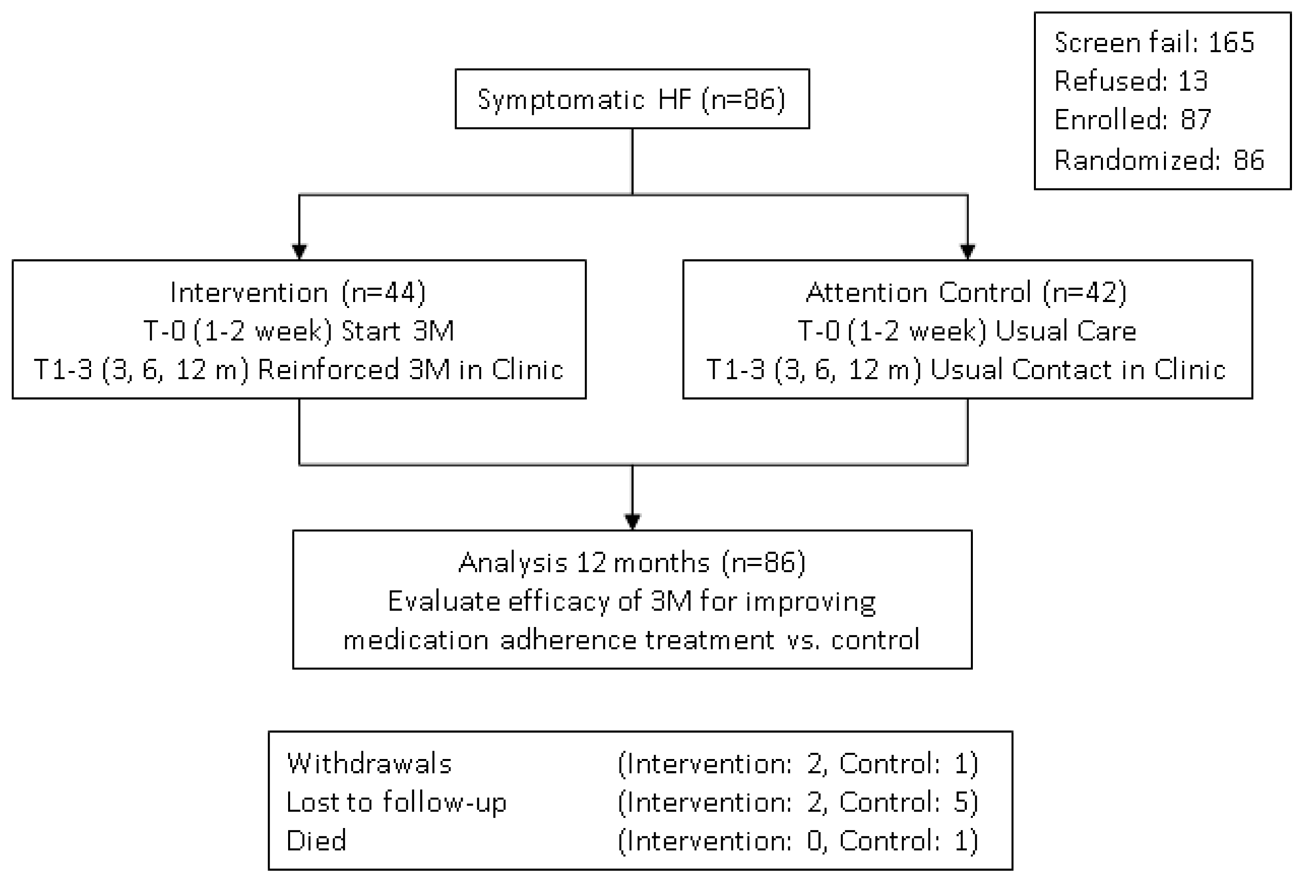
CHIME 3-M randomized design.

A total of 265 patients were screened for risk of poor adherence. Of these, 62% (n=165) received an MMAS 8-item score ≥6 and were ineligible for participation. Another 13 patients refused to participate after screening, and 1 withdrew prior to initiating the intervention, resulting in 86 patients randomized to intervention (n=44) or attention control (n=42) (Figure 1). The average age of the participants was 60 years (standard deviation [SD]±11.58), and the majority were black (n=57 [66%]), male (n=55 [64%]), married (n=44 [51%]), and had completed an 8th grade education (n=58 [67%]). Demographic characteristics including age, race, educational level, and indicators of disease severity were not different between treatment groups; however, the attention control group had significantly fewer women and fewer patients living alone (Table 3). All analyses were adjusted for these differences.

**Figure 2:**
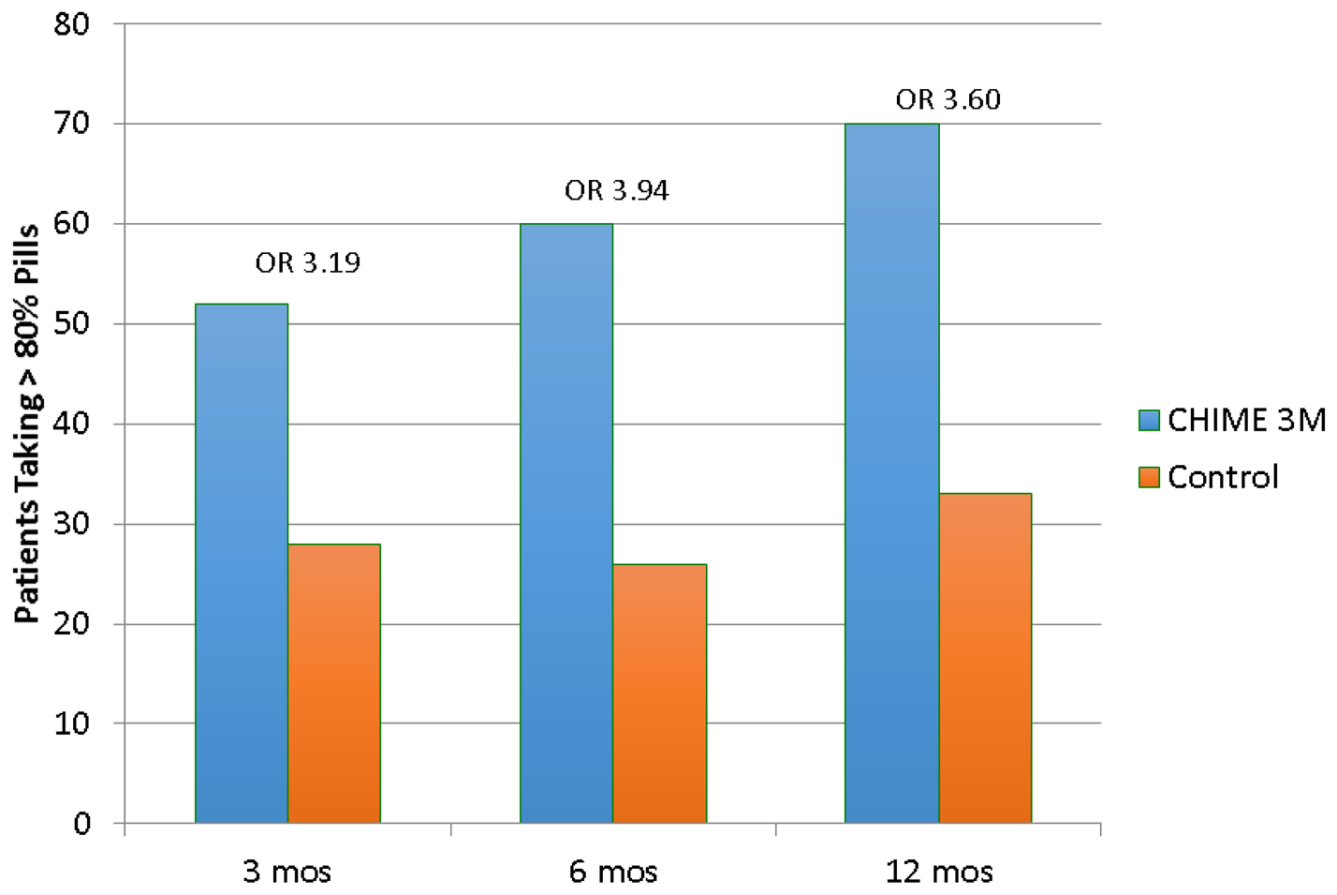
Medication adherence by nurse-assessed pill counts over time.

The primary endpoint of medication adherence was significantly improved in the intervention group as measured by nurse-assessed pill counts (Figure 2). Pooled over all time points, patients receiving the intervention were almost 4 times as likely to be adherent to medications as compared with those in the attention control group (odds ratio [OR] 3.92, t=3.51, p=0.0007).

**Figure 3:**
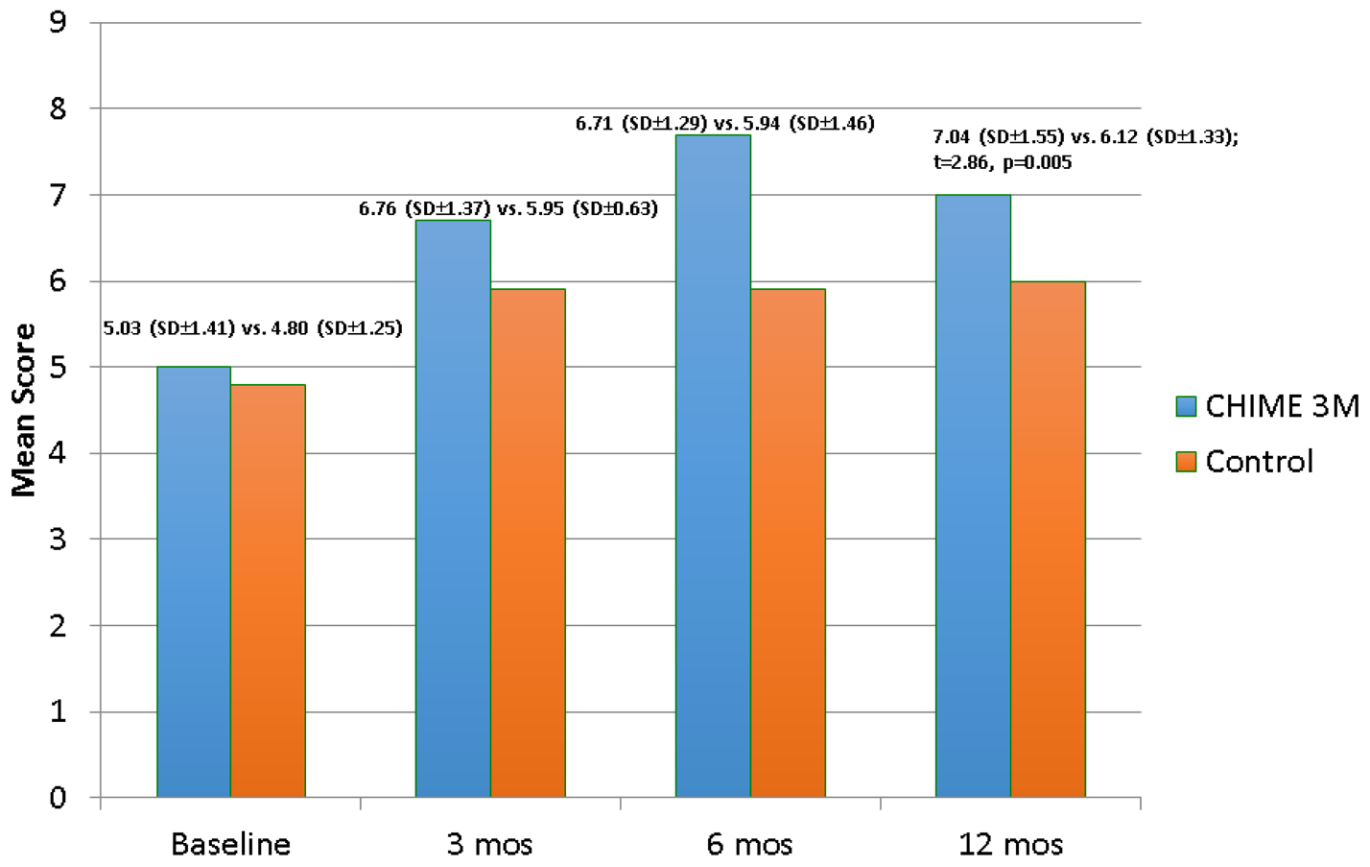
Medication adherence by Morisky Medication Adherence Scale (MMAS 8-item) scores over time.

The patient-reported adherence measure, MMAS 8-item, also indicated significantly higher adherence in the intervention group as compared with the attention control group pooled over all time points. The main effect of the intervention was statistically significant (t=2.86, p=0.0051), as was the main effect of time (t=6.75, p<.0001) in the mixed longitudinal model. Additionally, the time-by-intervention group interaction was not statistically significant and removed from the final model. Adjusted for the influence of time, MMAS 8-item mean scores were 6.10 for the CHIME 3-M and 5.70 for the control groups (Figure 3).

**Figure 4:**
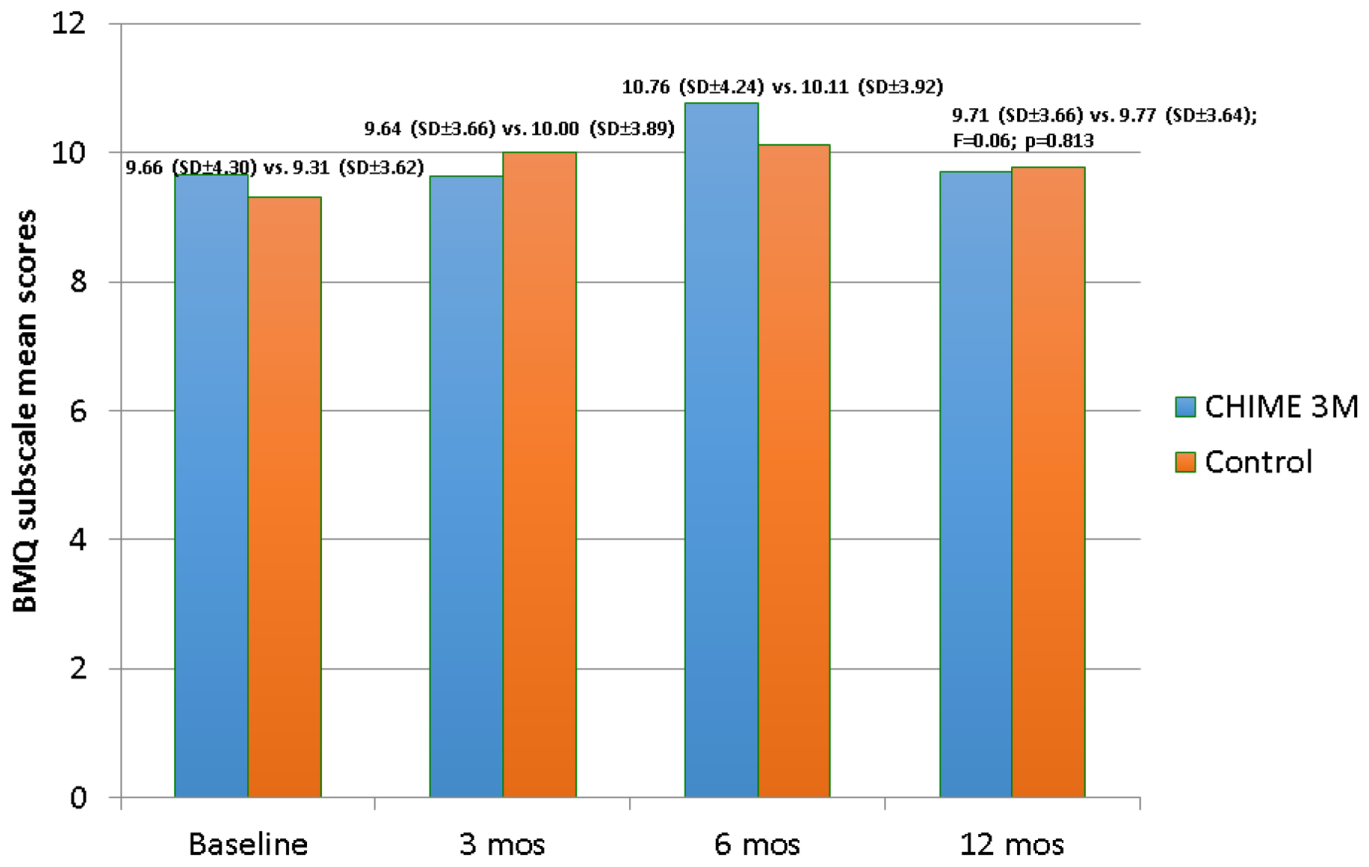
Belief in Medications Questionnaire trends over time.

The hypothesized mechanism of the intervention, improved meaning associated with the medication regimen, was measured by the BMQ. Patients in the intervention group showed a positive trend in subscale scores reporting higher perceived “*necessity* of medications” over time as compared with participants in the attention control group (Figure 4). Likewise, participants in the intervention group reported lower perceived “*concerns* with medications” subscale scores at 12 months as compared with those in the attention control group (F=4.29, p=0.04).

Patient-reported symptom frequency was similar in both groups at baseline and did not diverge between groups over time (monthly mean=20 episodes, SD 4.3). In contrast to the attention control group, however, the intervention group was asked to use the symptom-response plan to record timely and appropriate responses to symptom exacerbation. At each time point, the ratio of symptom experience to appropriate response was high (mean episodes=20, appropriate response=18; proportion of appropriate responders at each time point=75%). Though this study was not powered to evaluate readmission or emergency department use, the strategy yielded important qualitative data about patient decision-making that will be used in future work.

The nurse took field notes about the symptom episodes associated with hospital readmission and emergency department use. The most frequently reported reasons for using the emergency department included (in descending order of frequency): 1) symptom and side effect experiences were too overwhelming; 2) telephone contact with primary care provider advised me to go to the emergency department; and 3) the caregiver insisted on a hospital visit. The most frequent patient-reported reason for unplanned health resource use was the perceived need for provider evaluation, expressed as, “I don’t want to talk about it on the phone; I just want to be seen.”

## DISCUSSION

In this proof-of-concept study, an intervention that uses meaning-associations to coach patients through symptom changes and illness progression over the course of a year improved medication adherence by almost 4-fold in a cohort of high-risk patients with HF. Interventions of this type have not previously been evaluated in chronic HF, and yet, in the context of chronic illness with frequent symptom exacerbation, meaning-association interventions may be valuable adjuncts to the compendium of therapeutic interventions. The CHIME 3-M intervention facilitates recognition of symptom-triggers and coaches patients to develop meaningful associations between symptoms and prescribed medications. These associations are developed based on the patients’ expectations and beliefs about prescribed medications, their understanding of the need for the medications, and the perceived importance of the medication regimen for long-term outcomes, despite the work and complexities presented by the regimen for everyday life. Facilitating meaningful associations is hypothesized to improve the ability of the patient to decide for themselves that the overall benefit of taking a complex medication regimen for life outweighs the burdens imposed by doing so. Secondarily, facilitating meaningful association is hypothesized to be a useful tool that can be used in concert with a feasible symptom-response plan to manage one’s illness.

These findings represent proof-of-concept for a new genre of adherence intervention; one that integrates meaningful associations with medications through provider feedback about medication beliefs together with external behavioral drivers (toolkit for medication-taking reminders, physiologic self-monitoring, and health system supported follow-up calls and visits) to improve medication adherence. In patients with HF classified as being at high-risk for poor adherence to medications, these results suggest an avenue to improving medication adherence that has greater magnitude and persistence than others previously reported in the literature.

### Belief in medications and why reminders fall short

The intervention was designed to strengthen patient beliefs in the need for evidence-based medications for HF, and to lessen concerns about taking these medications for a lifetime. Our findings add empirical validity to the foundational work by Robert Horne, in which beliefs were first shown to be stronger predictors of adherence to medications than either clinical or sociodemographic factors.^34^ Across 4 different chronic illness populations (n=394), Horne and colleagues demonstrated the inverse relationship between concerns about the medication and the perceived benefit or “necessity” for taking medications. In the present study, the work of Horne is expanded to an intervention that can be scaled and used in clinical practice to support the patient-provider partnership.

In our study, scores on the BMQ corroborated the results on medication adherence. Our findings show that higher adherence is associated with cognitive perceptions of medications in which patients report lower levels of concern and higher levels of perceived necessity. Horne referred to this as the “cost-benefit” relationship; the risk that patients either overtly or subconsciously assign to prescribed medications, which influences medication-related decision making and pill-taking behaviors. Despite the importance of the meaning or beliefs that patients associate with prescribed medications, their perceptions are often not discussed with providers for reasons such as the patient’s desire to please and not disappoint the prescribing provider; the patient feeling their concerns may be trivialized or unwarranted; and providers neglecting to ask patients about sensitive issues such as the perceived need to take the medication.^35,36^

In an effort to encourage patient-provider communication, interventions that address “shared decision making” use commonly negotiated goals along with a core set of standard activities including establishing a relationship with a designated provider who has regular contact with the patient; easy access to a nurse; and reminders for medications, refills, and appointments.^25,37^ The CHIME 3-M intervention also incorporates shared decision making about medication. The nurse listened and encouraged discussion from the patient’s view of the “cost-benefit” for each medication, assessed necessity and concern, and allowed the patient to describe concerns that might affect adherence. These concerns could then be discussed and shared goals could be established more openly and honestly. For example, the timing or dose of diuretics might be adjusted to accommodate daily outings or a costly brand-name medication might be exchanged for a generic.

### The interaction of symptoms and belief

The most common expectation that patients with HF have is that their medications will control symptoms.^38^ When symptoms like fatigue and orthopnea fail to improve, patients have been found to lose confidence in and discontinue use of medications.^26,27^

In the present study, use of the symptom-response plan over the 12-month follow-up period provided an additional indicator of the efficacy of the intervention. Patients enrolled in the study were known to be non-adherent, and therefore highly likely to disengage from care and care provider support. Yet the core component of CHIME 3-M discussions about the meaning of the medications in everyday life was associated with not only sustained belief in the medications, but also sustained use of the agreed upon symptom-response plan for 12 months. These opportunities for communication may provide patients and providers time to address perceptions about medications that contribute to intentional non-adherence.^39^

### Implications for practice

The CHIME 3-M intervention offers a low-intensity, low-cost intervention built on the sustainable concept of internally motivated incentives. The average number of 6 calls per person per year (range, 4–16), at a cost of approximately $7.50 per call (nurse wage x time of the average call over 1 year), represents a substantial value for health systems, many of which currently sustain a loss on HF readmissions. By discussing everyday life, symptom patterns, and medications, the patient is able to partner with the provider to make medication-taking a meaningful aspect of self-care in chronic illness.

The principle behind combining a discussion of the lived experience of medication-taking and a skill-based intervention using calendar-set goals, scales, and pill boxes is to integrate the work of medication-taking with life and health. Medication-taking is framed as a daily health achievement, one that enables future goals such as daily activities or travel to be with family, to be attained over time.

This study has a number of limitations. The intervention was tested in a high-risk group of patients, both in terms of NYHA class (III IV) as well as adherence risk, who had recurrent symptom presentations and may not be representative of the general population of patients with HF. Yet the effect of the intervention in improving adherence by 2 independent measures persisted over time, lending credence to the notion that development of “meaning” associated with medications in chronic illness may be valuable and may hold promise for further development of a generalizable intervention for improving adherence in the larger population.

CHIME 3-M is built on the 3 core components of identification of medication goals, facilitation of medication-symptom associations, and use of a symptom-response plan. And yet the outcomes at 1 year, even given the inherent limitations of size, power, and generalizability, suggest that a new core component (beliefs and meaningful association with medications) can be feasibly implemented in high-risk patients, and may be effective in improving long-term medication adherence. The efficacy of this new component in a proof-of-concept study, testing an otherwise commonplace intervention of reminders, frequent follow-up, and scheduled patient-provider interaction, signifies the potential importance of “meaning” embedded in routinized chronic medication-taking regimens.

### Conclusions

The goal was to develop an intervention that would support medication-taking, even in the context of recurrent symptoms, by supporting patients using a combination of symptom-associated triggers, internal cognitive and external social supports, and by developing meaningful patient perceptions of the value of the medication regimen itself.

## Figures and Tables

**Table 1:** CHIME 3-M outcome measures

CHIME 3-M Component	Outcome Measure	Reliability/Validity
1. “Medication”	Medication adherence	

Patient-reported measure	MMAS 8-item	8-item, 5-point Likert scale instrument designed to predict risk for poor medication-taking habits. Cronbach’s alpha, this HF cohort 0.62–0.72; concurrent and predictive validity are strong
Observed measure	Nurse assessment of medications taken (pill count evaluation)	

2. “Meaning”	Belief: Necessity/Concerns	

Patient-reported measure	BMQ	The BMQ-Specific comprises 2 5-item factors assessing beliefs about the necessity of prescribed medication (Specific- Necessity) and concerns about prescribed medication based on beliefs about the danger of dependence and long-term toxicity and the disruptive effects of medication (Specific-Concerns). Cronbach’s alpha 0.76 on both subscales for cardiac patients; concurrent and predictive validity are strong
Observed measure	Field note counts loss of belief and concerns regarding medication discontinuation	

3. “Me”	Patient’s symptom profile	

Observed measure	Symptom response frequency and use of response plan	

MMAS 8-item, Morisky Medication Adherence Scale; BMQ indicates Belief in Medications Questionnaire.

**Table 2:** CHIME 3-M Symptom-Response Plan

Component	Procedure The patient, nurse, and caregiver together will:
WHAT: Define the patient’s typical symptom trigger.	Use the patient’s symptom profile from the “Me” component of the 3-M intervention to identify the most common and also the most frequent early symptom (these may be the same).
WHEN: Define the immediacy with which the patient should activate the symptom response plan once symptoms occur.	Identify a commonly agreed upon timeframe for response to symptom onset; ensure that the patient and caregiver (if applicable) agree on a communication plan for reporting symptoms and an initial versus persistent symptom trigger
WHO: Define the resources to active at the time of symptom onset.	Identify who should be called (people and/or community services) and determine the order of priority in which these calls should be made.
HOW: Define the correct phone numbers for each contact	Identify these on the 3M calendar *Symptom-Response Plan* page; post the response plan contact list in a visible and accessible location at home.

Formulation of the symptom-response plan was developed in collaboration with coauthors (IE) as previously published and refers specifically to the patient perspective on the plan of action, timing, and transportation.

**Table 3:** Baseline characteristics by treatment group (n=86)

Characteristic	Intervention (n=44)	Attention Control (n=42)	Total	P Value
Age, yrs				0.91
Mean (±SD)	60.24 (11.60)	59.94 (11.52)		
Median (IQR)	62.09 (17.79)	59.65 (18.13)		
Sex				0.03
Male	23	32	55	
Female	21	10	31	
Race				0.36
White	12	16	28	
Nonwhite	32	26	58	
Level of education				0.65
0–12 years	31	27	58	
≥13 years	13	15	28	
Diabetes	25 (58.14)	25 (62.50)	50 (60.24)	0.685
Hypertension	37 (86.05)	32 (80.00)	69 (83.13)	0.462
Lives alone				0.04
Yes	12	4	16	
No	32	37	69	
Marital status				0.14
Married	19	25	44	
Not married	25	17	42	
ACE-I prescribed	21	20	42	
B-blocker prescribed	38	31	69	
Diuretic prescribed	39	31	70	
Aldosterone antagonist	17	18	35	

Values are expressed as number (%), unless otherwise indicated.

IQR indicates interquartile range; SD, standard deviation.

## Summary of key points

1. The CHIME 3-M intervention improved medication adherence by almost 4-fold among patients with HF at high risk for medication non-adherence.
2. In HF, as in many chronic illnesses, persistent symptoms are an inherent component of the illness. Symptoms that exacerbate and escalate with increasing frequency and intensity over time are primary reasons for loss of belief in medications. Equally prevalent, however, among reasons to stop medications is the cessation of symptoms (causing patients to feel the medication is no longer necessary).
3. Interventions that remind a person to take medications (if medications are perceived as not beneficial, not necessary, or posing a concern) are an ineffective long-term intervention. As a result, reminder interventions do not address “intentional non-adherence” or the patients’ perception that the medications are “meaningless” for their daily life or long-term outcomes.
4. The CHIME 3-M intervention integrates 3 components (Medication information: clarification of mechanisms; Meaning: “right-sizing” expectations patients have for medications and their beliefs in medications as they pertain to HF; and Me: individual symptom recognition and response patterns, as these are associated with medications) to help patients reframe the need for medications and re-appropriate their concerns about taking HF medications for a lifetime.
5. The CHIME 3-M intervention is unique because it provides an infrastructure to regularly address perceived necessity and concern with patients and iteratively reframe a patients’ cognitive (representation) of prescribed medications. The mechanism of such an approach may provide a basis for a closer partnership between providers and patients for more successful mediation-taking behaviors.
